# The Medical Bulletin Visiting List

**Published:** 1891-01

**Authors:** 


THE MEDICAL BULLETIN VISITING LIST, OR PHYSICIAN'S CALL RECORD. Arranged upon an Original and Convenient Monthly and Weekly Plan for the Daily Recording of Professional Visits. F. A. Davis, Medical Publisher and Bookseller, 1231 Filbert street, Philadelphia, Pa.

This visiting list differs from all others in its make-up, and possesses some excellent advantages. It is published in three sizes, at the following prices: No. 1, $1.25; No. 2, $1.50; No. 3, $1.75. Its preliminary matter is of a valuable character, and serves to refresh the memory on many useful points for bedside reference.

